# Radioguided localization of recurrent lymph nodes in differentiated thyroid cancer - Where are we now?

**DOI:** 10.20945/2359-3997000000079

**Published:** 2018-10-01

**Affiliations:** 1 Universidade de São Paulo Universidade de São Paulo Faculdade de Medicina Departamento de Radiologia e Oncologia São Paulo SP Brasil Disciplina de Medicina Nuclear, Departamento de Radiologia e Oncologia da Faculdade de Medicina da Universidade de São Paulo (FMUSP), São Paulo, SP Brasil

Controlling loco-regional recurrence is an important goal for patients with differentiated thyroid cancer (DTC). A precise localization of suspected lesions is critical for patients with recurrence following a therapeutic neck dissection, as reoperations present increased morbidity. A focused excision may be justified, and marking of non-palpable lesions can help optimize surgical approach and increase tumor detection and removal.

Radioguided occult lesion localization (ROLL) was initially described for breast tumors, and is based on the intra-operatory guidance by a hand-held gamma-probe that detects radiation emitted from ^99m^Tc-macroaggregated albumin (^99m^Tc-MAA), previously injected into the lesion during an ultrasound or mammography. The excellent results of ROLL for non-palpable breast lesions, as evidenced by systematic reviews and meta-analysis (1), lead to its use in other clinical situations.

Radioguided procedures are also performed with radiopharmaceuticals smaller than ^99m^Tc-MAA, capable of following the physiological lymphatic drainage from the injection site, and to identify and guide sentinel lymph node (SLN) biopsy. For patients with DTC, a recent systematic review proposes that lymphoscintigraphy could be used to guide compartment-oriented lymphadenectomy but not to restrict biopsy of SLNs only (2). Evidently, SLN biopsy is a procedure for initial staging and not an option for post-surgical restaging or recurrent disease localization.

In recent years, the interest in ROLL procedures to guide metastatic lymph node (LN) excision in DTC has increased. In this issue of *Archives of Endocrinology and Metabolism* (AE&M), Cerit and cols. present their experience using intralesional injection of ^99m^Tc-MAA under ultrasound guidance for radioguided resection of lymph nodes in 11 patients with recurrent or persistent DTC (3). The authors used the term Guided intra-Operative Scintigraphic Tumor Targeting (GOSTT), originally proposed to include the use of different tracers, multimodality equipments, and 3D navigational systems (4).

Although several radiopharmaceuticals are described to localize recurrent/ persistent metastatic LN in DTC, including tumor-avid tracers such as ^99m^Tc-sestamibi (5), ^18^F-FDG, and iodine-131 (6), Cerit and cols. use the classic concept of ROLL. Injection of ^99m^Tc-MAA into the suspicious lymph nodes was performed under ultrasound (US) monitoring, 1 to 2 h prior to surgery. A diagnosis of DTC metastasis had been previously established via US-guided fine needle aspiration biopsy (FNAB) and FNAB-Tg washout level. So, it is not surprising that all of the 26 marked LNs revealed DTC metastasis on histopathologic examination.

It is also of note that metastasis were present in 14 from the total of 85 additional non-marked LNs removed (16%). In the study of Gulcelik, including the use of ROLL for locoregional metastasis in 20 DTC patients, 36 out of 56 (64%) suspicious non-marked lesions were metastatic (7). Detection of metastasis in non-marked adjacent LNs points to the fact that disease may also be present in other LNs that were not removed. Even if the initial purpose of the surgery is not to eliminate all micrometastasis, it is not clear the impact of residual disease in future recurrences and what will be the role of radioiodine therapy or other systemic therapies in this scenario.

Regarding recurrence after ROLL, the authors state that even subcentimetric lesions were excised and local control of the disease was achieved in all patients after a median follow-up of 30 months (a 2nd ROLL was necessary in one case and 3 patients presented with lung or mediastinal metastasis during follow-up). Borsò and cols. (8) also found 33% to 40% recurrence rate after a follow-up of 29 months in 32 patients with DTC submitted to metastatic LN ROLL.

These findings do not invalidate the use of ROLL to improve identification of suspicious lesions, and possibly to increase the completeness of resection in a difficult surgical field, with the intention of improving prognosis of patients with recurrent DTC. However, despite the encouraging results, at this moment the limited number of patients and follow-up interval do not allow a clear definition of ROLL impact in longterm outcomes and clinical recurrence of DTC patients.

Available data suggests that radioguided dissection is safe and feasible in patients with loco-regional recurrence from DTC, and may be an alternative for controlling DTC LN recurrence with low morbidity. The procedure requires an intensive coordination among different specialties: surgeon, radiologist, nuclear medicine physician, and pathologist; and careful planning is required by the institutions that intend to adopt it.
